# Seventeen-Year Clinical Analysis of Acute Primary Angle-Closure Glaucoma at a Single Japanese Tertiary Center

**DOI:** 10.7759/cureus.112749

**Published:** 2026-07-15

**Authors:** Nozomi Tanaka, Etsuko Terao, Yasuko Fujisawa, Yuki Nagata, Ryota Aoki, HIroyuki Wakuda, Shunsuke Nakakura

**Affiliations:** 1 Ophthalmology, Saneikai Tsukazaki Hospital, Himeji, JPN

**Keywords:** acute primary angle-closure glaucoma, intraocular pressure, paralytic mydriasis, phacoemulsification, visual acuity

## Abstract

Purpose: Acute primary angle-closure glaucoma (APAC) is a sight-threatening emergency that requires immediate intervention; however, there are few reports regarding its therapeutic efficacy and complications. We analyzed 17-year clinical characteristics, surgical outcomes, intraocular pressure (IOP) trajectories, visual acuity (VA), and complications in APAC at a Japanese tertiary center.

Study design: This is a retrospective, single-center, observational cohort study.

Methods: Records of 210 patients (229 eyes) with APAC between June 2009 and September 2025 were reviewed. Demographics, presenting IOP, biometric parameters, surgical procedure, VA, and postoperative complications were evaluated. IOP at baseline, postoperative day 1 (POD1), one week, one month, and three months was compared using the Wilcoxon signed-rank test with Bonferroni correction. Onset distribution was assessed using chi-square goodness-of-fit tests.

Results: Mean age was 73.2 ± 10.0 years; 68.6% were female. Mean presenting IOP was 53.2 ± 15.4 mmHg. Initial phacoemulsification with intraocular lens implantation (PEA+IOL) was completed in 93.0% of cases. IOP decreased to 16.1 ± 9.9, 15.1 ± 7.3, 14.2 ± 4.7, and 13.9 ± 3.4 mmHg at POD1, one week, one month, and three months, respectively (all corrected p < 0.001). Mean logMAR VA improved from 1.26 ± 0.99 to 0.31 ± 0.87. Paralytic mydriasis occurred in 43.2%, and 26.6% required additional glaucoma treatment. Onset showed no significant deviation from uniform distribution monthly (p = 0.095) or seasonally (p = 0.895).

Conclusion: Initial PEA+IOL produced sustained IOP reduction and improved VA in Japanese patients with APAC. APAC onset was evenly distributed throughout the year. The high rates of paralytic mydriasis and additional treatment in more than one-quarter of eyes highlight the importance of long-term follow-up.

## Introduction

Acute primary angle-closure glaucoma (APAC) is a sight-threatening ophthalmic emergency characterized by a sudden, marked elevation of intraocular pressure (IOP) due to complete iridotrabecular contact [1,2]. Patients typically present with severe ocular pain, headache, blurred vision, corneal edema, and a mid-dilated fixed pupil. Without prompt treatment, irreversible optic nerve damage and vision loss can occur within hours.

The epidemiology of APAC shows a well-established ethnic gradient. Asian populations carry a substantially higher burden, with crude incidence rates of approximately 10-12 per 100,000 persons per year in Singapore and Hong Kong, compared with 3.9-4.1 per 100,000 per year in European populations [2]. The disease preferentially affects elderly women with hyperopic, anatomically predisposed eyes, a pattern consistently observed across multiple cohort studies [3,4] and reflected in the present series.

Historically, laser peripheral iridotomy (LPI) following medical IOP reduction was the cornerstone of APAC management. However, compelling evidence from randomized controlled trials, including the landmark studies by Lam et al. [5] and Husain et al. [6], has established that primary phacoemulsification with intraocular lens implantation (PEA+IOL) achieves superior IOP control and reduces the requirement for long-term glaucoma medications [7-10]. The primary objective of this study was to investigate in detail the clinical background of patients with APAC and to evaluate the effect of initial treatment with PEA+IOL for APAC. The secondary objective was to assess complications following APAC treatment, with particular attention to the incidence of postoperative paralytic mydriasis.

## Materials and methods

Study design and participants

This was a single-center retrospective observational cohort study. Medical records of all patients referred to Saneikai Tsukazaki Hospital with suspected APAC between June 2009 and September 2025 were reviewed. APAC was defined as an acute episode of angle closure with IOP ≥ 30 mmHg, consistent presenting symptoms (ocular pain, visual blurring, halos), a closed drainage angle on gonioscopy, and corneal edema or glaukomflecken on slit-lamp examination [1,2].

A total of 277 eyes were initially screened. Forty-eight eyes were excluded for the following reasons: 36 eyes in which the APAC attack had spontaneously resolved or had been medically released at the referring facility before presentation, and 12 eyes with a final diagnosis of secondary angle closure (neovascular glaucoma or exfoliation glaucoma). The final analytic cohort comprised 210 unique patients (229 eyes).

This study was conducted in accordance with the tenets of the Declaration of Helsinki. The study protocol was approved by the Institutional Review Board of Saneikai Tsukazaki Hospital (No. 2601042), with explicit authorization to retrospectively analyze clinical records dating back to June 2009. Given the retrospective nature of the study, the requirement for written informed consent from individual patients was waived by the IRB, and an opt-out procedure was provided via hospital website notification.

Outcome measures

Primary outcomes included IOP at each postoperative time point (baseline, POD1, one week, one month, three months) and logMAR VA at baseline and at the final follow-up visit. Secondary outcomes included surgical procedure distribution, central corneal thickness (CCT), axial length (AL), anterior chamber depth (ACD), onset-to-presentation interval, and postoperative complications (paralytic mydriasis and additional glaucoma treatment).

Paralytic mydriasis was assessed at the final follow-up examination based on slit-lamp biomicroscopy findings, supplemented by photographic documentation when available. In unilateral cases, paralytic mydriasis was diagnosed when the affected pupil exceeded the contralateral pupil diameter by an unequivocal interocular difference under standardized examination lighting. In bilateral APAC, paralytic mydriasis was diagnosed when the pupil failed to constrict below approximately 4 mm under slit-lamp illumination, consistent with iris sphincter dysfunction. Cases in which the medical record did not contain sufficient information for this judgment were classified as “unknown.”

Additional glaucoma treatment was defined as the introduction of any topical IOP-lowering medication or the performance of any additional glaucoma procedure (trabeculectomy, trabeculotomy, or tube shunt surgery) during the postoperative follow-up period.

Visual acuity conversion

Visual acuity (VA) was recorded in its original units (decimal or qualitative descriptors) and subsequently converted to logMAR for the calculation of means and standard deviations. The following conventions were applied: no light perception (NLP) = 4.0, light perception (LP) = 3.0, hand motion (HM) = 2.3, counting fingers (CF) = 2.0, and decimal VA = −log10(VA), as described by Holladay (1997) [11].

Biometric measurements

CCT was measured with a non-contact specular microscope (CEM-530, NIDEK Co., Ltd., Gamagori, Japan) or by anterior-segment optical coherence tomography (CASIA2, Tomey Corporation, Nagoya, Japan) when corneal edema precluded specular microscopy. AL and ACD were measured with an optical biometer (IOLMaster 500 or 700, Carl Zeiss Meditec AG, Jena, Germany) when media clarity permitted and supplemented by immersion ultrasonography in eyes with dense corneal edema.

Surgical intervention

In our institution, board-certified ophthalmologists with experience of at least approximately 300 cataract surgeries select PEA+IOL as the first-line procedure for APAC. Once a diagnosis of APAC was confirmed, intravenous hyperosmotic agents and topical IOP-lowering medications were administered. After lens-power calculation and pharmacological mydriasis, PEA+IOL was attempted, in principle, on the same day. When intraocular lens implantation was not technically feasible at the time of the initial surgery, or when PEA+IOL itself was deemed not feasible and LPI was performed as the initial intervention instead, secondary IOL implantation was subsequently carried out within two weeks if necessary.

Statistical analysis

Descriptive statistics are reported as mean ± standard deviation (SD). Because IOP distributions deviated significantly from normality at baseline and all postoperative time points (Shapiro-Wilk p < 0.01 at baseline and three months; p < 0.001 at POD1, one week, and one month), comparisons of postoperative IOP at each time point against baseline were performed using the Wilcoxon signed-rank test, restricted to paired observations with complete data at both time points. Bonferroni correction was applied to account for the four pairwise comparisons (corrected α = 0.0125). To address the potential intra-patient clustering in 19 patients with bilateral involvement, a sensitivity analysis was performed restricting the dataset to one eye per patient (the worse-presenting eye); results were directionally identical and are not separately reported. The chi-square goodness-of-fit test was used to assess whether the monthly and seasonal distributions of APAC onset deviated significantly from a uniform distribution. A two-sided p-value < 0.05 was considered statistically significant; values below 0.001 are reported as p < 0.001. All statistical analyses were performed using Python (SciPy library, version 1.11; Python Software Foundation, Beaverton, OR, USA).

## Results

Patient demographics

**Figure 1:**
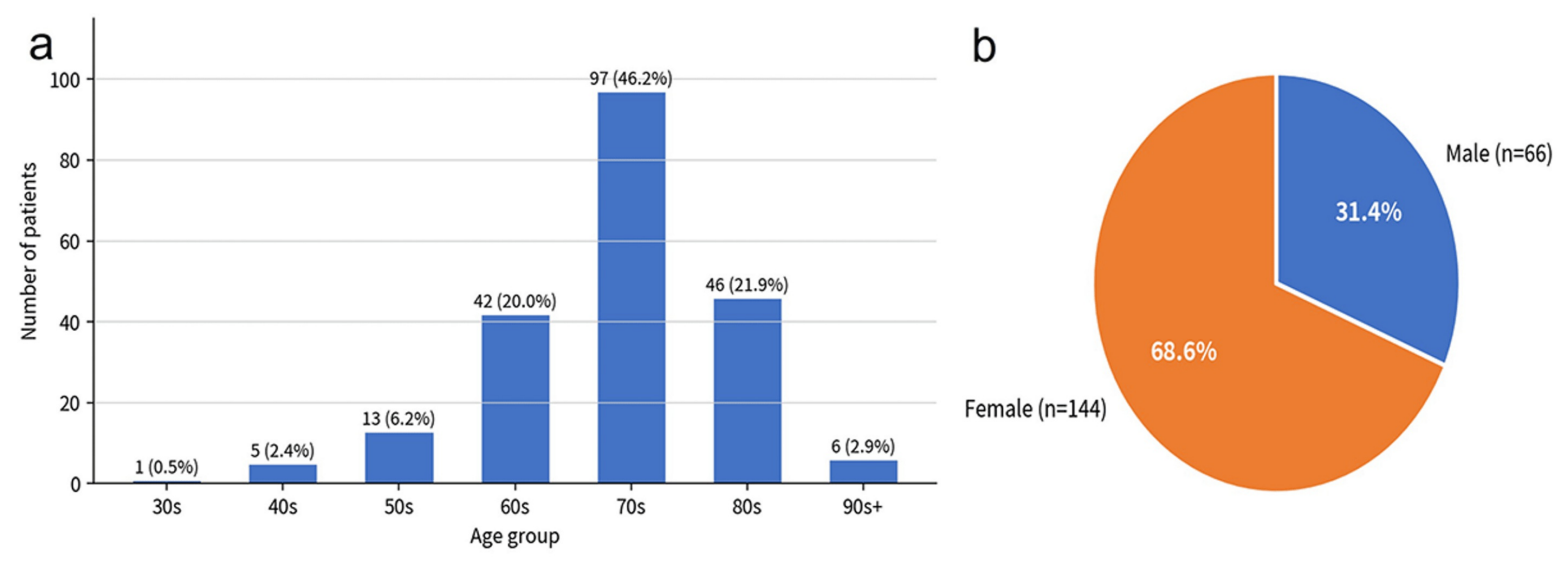
Age and sex distribution of APAC patients a. Age distribution of 210 unique APAC patients. Mean age 73.2 ± 10.0 years. The majority of patients were in their 70s (n = 97, 46.2%). b. Sex distribution. Female patients comprised 68.6% of the cohort (n = 144), consistent with the known female predominance of APAC in Asian populations. APAC: Acute primary angle-closure glaucoma

Of 277 eyes initially screened, 48 eyes were excluded (36 with spontaneously or medically resolved attacks at the referring facility; 12 with neovascular glaucoma or pseudoexfoliation glaucoma). The final cohort comprised 210 unique patients (229 eyes); 19 patients (9.0%) had bilateral involvement. Mean age at diagnosis was 73.2 ± 10.0 years (range 39-96 years), with the majority of patients in their seventh and eighth decades (Figure 1a). The sex distribution revealed a marked female predominance, with 144 female (68.6%) and 66 male (31.4%) patients (Figure 1b). These findings are consistent with the well-established epidemiological profile of APAC in East Asian populations [2-4].

Symptom onset to presentation

**Figure 2:**
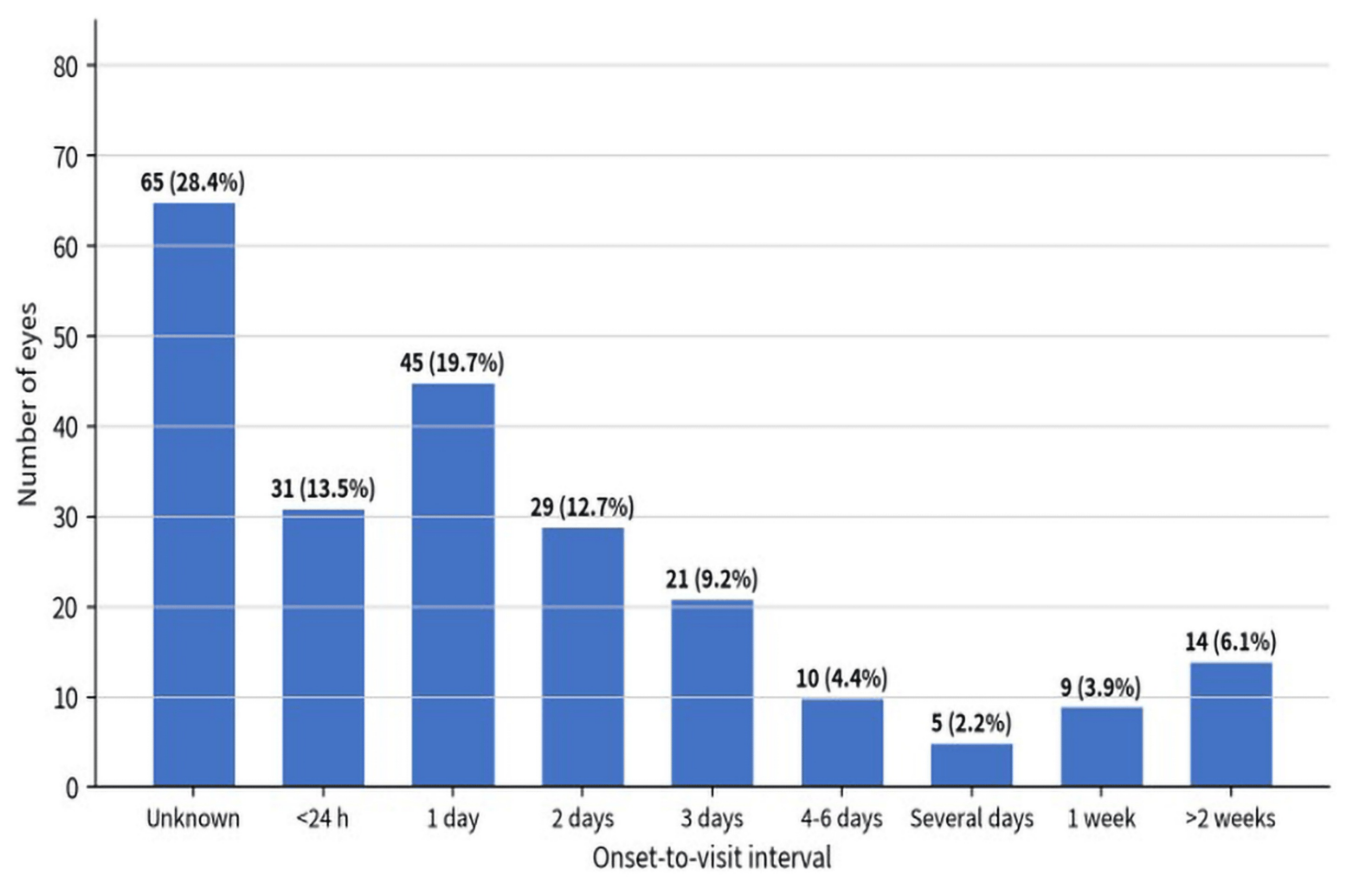
Distribution of symptom onset-to-presentation intervals Among documented cases, the most common interval was one day (45 eyes, 19.7%), followed by less than 24 hours (31 eyes, 13.5%) and two days (29 eyes, 12.7%).

The onset-to-presentation interval was documented in 164 of 229 eyes (71.6%), with the remainder classified as unknown (Figure 2). Among documented cases, the most common interval was one day (45 eyes, 19.7%), followed by less than 24 hours (31 eyes, 13.5%) and two days (29 eyes, 12.7%). Fourteen eyes (6.1%) presented after more than two weeks. The most frequently reported symptoms at presentation were ocular pain (46.3%), visual deterioration (22.7%), and headache (13.5%) [2].

Biometric parameters: CCT, axial length, and anterior chamber depth

**Figure 3:**
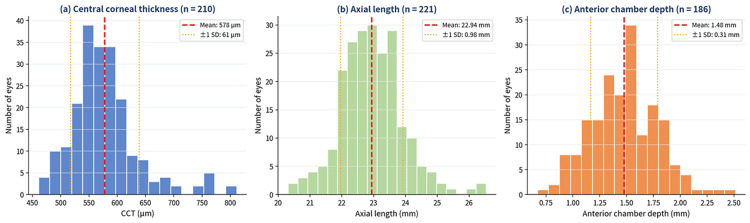
Biometric measurements at presentation (a) Central corneal thickness (n = 210 eyes), mean 578 ± 61 μm. (b) Axial length (n = 221 eyes), mean 22.94 ± 0.98 mm. (c) Anterior chamber depth (n = 186 eyes), mean 1.48 ± 0.31 mm. Both the short axial length and shallow anterior chamber reflect the anatomical predisposition to pupillary block in APAC eyes. APAC: Acute primary angle-closure glaucoma

CCT was available for 210 eyes, AL for 221 eyes, and ACD for 186 eyes (Figure 3). The mean CCT was 578 ± 61 μm (range 461-811 μm), somewhat above normative levels for Japanese adults (543 μm) [12], likely reflecting acute corneal edema from pressure-induced endothelial dysfunction in a subset of patients [1]. The mean AL was 22.94 ± 0.98 mm (range 20.31-26.53 mm), shorter than the normative AL of approximately 23.6-24.0 mm in Japanese adults [12]. The mean ACD was 1.48 ± 0.31 mm (range 0.67-2.51 mm), markedly shallower than normal values (3.21 mm) [12]. These short AL and shallow anterior chamber findings are consistent with the well-established anatomical predisposition to pupillary block and angle closure that characterizes APAC eyes [1,3,4].

Surgical procedures

**Figure 4:**
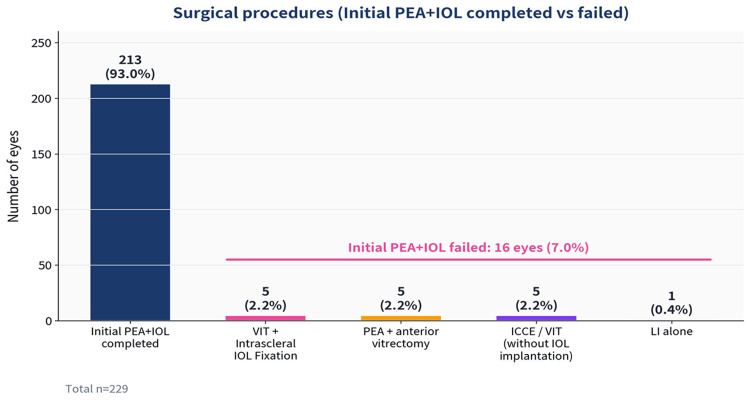
Distribution of surgical procedures Initial PEA+IOL was performed in 93.0% of cases. ICCE: Intracapsular cataract extraction; PEA: phacoemulsification; IOL: intraocular lens; VIT: vitrectomy; LI: laser iridotomy

Initial PEA+IOL with in-the-bag IOL implantation was completed in 213 eyes (93.0%), affirming its role as the standard of care (Figure 4) [5,6,8]. This category included three eyes (1.3%) that underwent concurrent goniosynechialysis (PEA+IOL+GSL) and three eyes (1.3%) that underwent concurrent posterior vitrectomy (PEA+IOL+posterior vitrectomy). Initial PEA+IOL was unsuccessful in 16 eyes (7.0%): posterior capsular rupture, zonular dialysis, or other intraoperative complications led to alternative procedures in 15 eyes, and intraoperative aborting due to severe corneal opacity led to LPI in one eye. Specifically, pars plana vitrectomy with intrascleral IOL fixation was performed in five eyes (2.2%), PEA with anterior vitrectomy in five eyes (2.2%), intracapsular cataract extraction with anterior or posterior in five eyes (2.2%), and LPI as the definitive alternative treatment in one eye (0.4%).

Intraocular pressure outcomes

**Figure 5:**
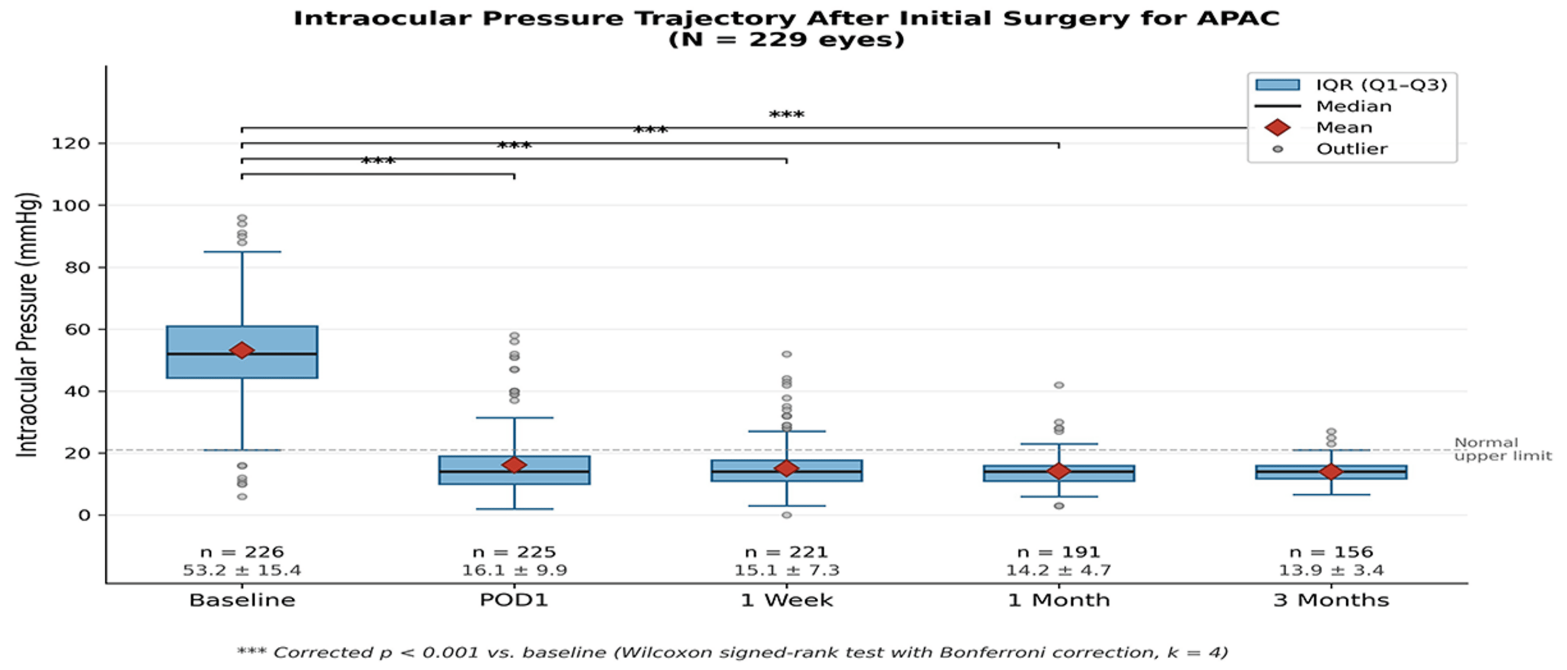
Box-and-whisker plots of IOP at baseline (n = 226), POD1 (n = 225), one week (n = 221), one month (n = 191), and three months (n = 156) Horizontal lines indicate medians; boxes represent Q1–Q3; whiskers extend from Q1 − 1.5 × IQR to Q3 + 1.5 × IQR; dots represent outliers. All postoperative time points were significantly lower than baseline (corrected p < 0.001, Wilcoxon signed-rank test with Bonferroni correction).

Mean presenting IOP was 53.2 ± 15.4 mmHg (n = 226). Following initial surgery, IOP declined dramatically at every assessed time point (Figure 5). Wilcoxon signed-rank testing with Bonferroni correction confirmed a highly significant reduction at all postoperative intervals (all corrected p < 0.001). At three months, mean IOP was 13.9 ± 3.4 mmHg (n = 156), a 73.9% reduction from baseline, with markedly reduced variability (SD 15.4 → 3.4 mmHg), reflecting both surgical efficacy [6,7] and resolution of post-attack inflammation.

Visual acuity outcomes

**Figure 6:**
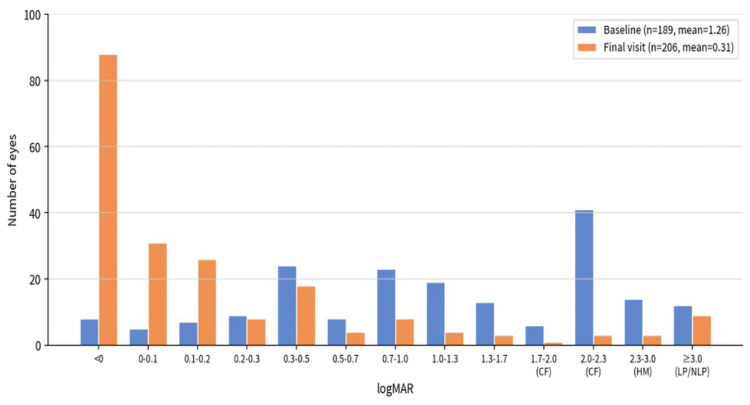
Visual acuity distribution at baseline (blue) and final visit (orange) expressed in logMAR intervals NLP: no light perception; LP: light perception; HM: hand motion; CF: counting fingers; VA: visual acuity. At baseline, the most common VA category was CF-equivalent (logMAR 2.0–2.3; 41 eyes, 21.7%); by the final visit, the modal category had shifted to decimal VA ≥ 1.0 (logMAR ≤ 0; 88 eyes, 42.7%)

Baseline logMAR VA was available for 189 eyes (mean 1.26 ± 0.99; Snellen equivalent approximately 0.05). At the final visit (206 eyes), mean logMAR VA improved to 0.31 ± 0.87 (Snellen equivalent approximately 0.49), representing a mean improvement of 0.95 logMAR units (~9-10 Snellen lines) [11]. At baseline, the most common VA category was CF-equivalent (logMAR 2.0-2.3; 41 eyes, 21.7%); by the final visit, the modal category had shifted to decimal VA ≥ 1.0 (logMAR ≤ 0; 88 eyes, 42.7%) (Figure 6).

Postoperative complications

**Figure 7:**
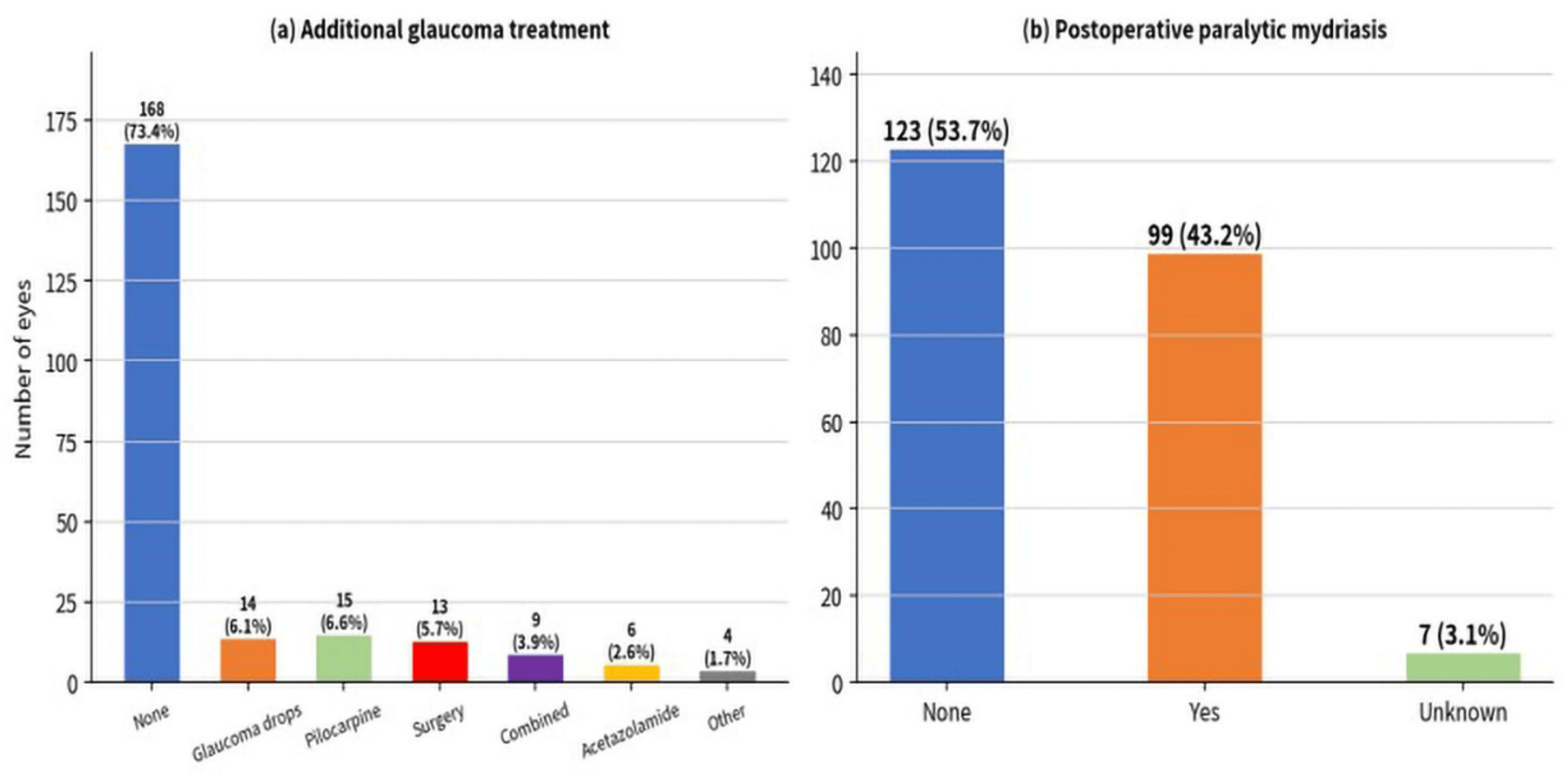
Postoperative complications (a) Distribution of additional glaucoma treatment categories (n = 229 eyes). (b) Incidence of postoperative paralytic mydriasis (n = 229 eyes).

Postoperative complications are summarized in Figure 7. Paralytic mydriasis was documented in 99 eyes (43.2%), with seven eyes (3.1%) classified as unknown and 123 eyes (53.7%) without documented paralytic mydriasis. When the analysis was restricted to eyes with definitive documentation (n = 222), the proportion with paralytic mydriasis was 99/222 (44.6%). This high rate likely reflects the severity of acute IOP elevation causing iris sphincter ischemia [1].

Additional glaucoma treatment was required in 61 eyes (26.6%). Among those treated, pilocarpine (14 eyes, 6.1%) was used in the early postoperative phase for IOP reduction and pupillary management, and prostaglandin analogs or other ocular hypotensives (14 eyes, 6.1%) were most common in the late postoperative phase. Thirteen eyes (5.7%) required additional surgical intervention (4 trabeculectomy, 1 trabeculotomy, and 8 IOL fixation). Four eyes (1.7%) received a sub-Tenon steroid injection for cystoid macular edema.

Seasonal and monthly distribution of onset

**Figure 8:**
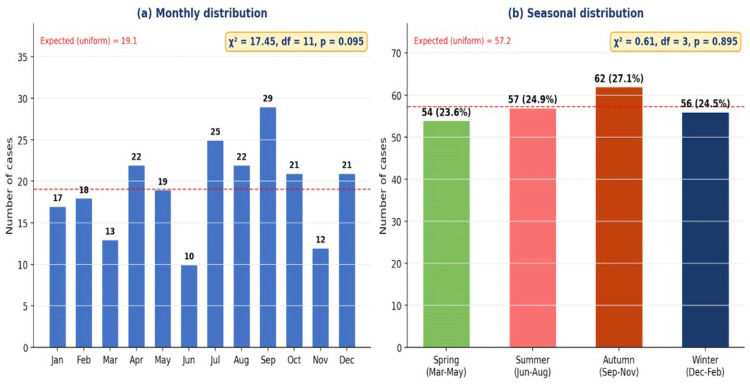
Temporal distribution of APAC onset (n = 229 eyes) (a) Monthly distribution; (b) seasonal distribution. Red dashed lines indicate the expected counts under a uniform distribution (19.1 per month and 57.2 per season). Chi-square goodness-of-fit tests showed no significant deviation from a uniform distribution either in the monthly (p = 0.095) or seasonal (p = 0.895) analysis. APAC: Acute primary angle-closure glaucoma

The temporal distribution of APAC onset across all 229 eyes was analyzed using the date of the initial visit (Figure 8). The number of monthly cases ranged from 10 (June) to 29 (September), with the highest occurrence in September (29 cases, 12.7%) and the lowest in June (10 cases, 4.4%). When grouped into seasons, autumn (September-November) accounted for 62 cases (27.1%), summer (June-August) for 57 cases (24.9%), winter (December-February) for 56 cases (24.5%), and spring (March-May) for 54 cases (23.6%). A chi-square goodness-of-fit test against a uniform distribution showed no statistically significant deviation either in the monthly distribution (χ² = 17.45, df = 11, p = 0.095) or in the seasonal distribution (χ² = 0.61, df = 3, p = 0.895). These findings indicate that, in our cohort, APAC onset did not significantly deviate from a uniform temporal distribution at the α = 0.05 level.

## Discussion

This 17-year retrospective analysis demonstrates that initial PEA+IOL is a safe and highly effective treatment for APAC in a Japanese tertiary care setting, producing significant and sustained IOP reduction with substantial visual recovery. Our findings are broadly concordant with published evidence [5-8] while providing additional information on postoperative complications, particularly the high rate of paralytic mydriasis, that is infrequently detailed in the existing literature. The initial PEA+IOL failure rate due to posterior capsular rupture or zonular dialysis was 6.6% (15 eyes) in our APAC cohort, excluding one eye in which LPI was performed after intraoperative aborting due to corneal opacity. This rate was substantially higher than the posterior capsule rupture rates of approximately 1.5%-3.4% reported for routine non-APAC cataract surgery, even among trainee surgeons [13,14], likely reflecting the increased technical demands imposed by corneal edema and zonular weakness characteristic of post-attack APAC eyes. We further compared surgeon experience (years since board certification) between the completed-surgery group (n = 213) and the failed-surgery group (n = 16); no significant difference was observed (11.2 ± 5.3 vs. 10.0 ± 5.5 years; p = 0.421, Mann-Whitney U test). Therefore, careful intraoperative management is required when performing initial PEA+IOL for APAC.

The demographic profile of our cohort is consistent with established epidemiological data for East Asian APAC populations [2-4]. The mean age of 73.2 years and female predominance of 68.6% reflect the anatomical risk factors, short AL, shallow anterior chamber, and thick crystalline lens, that predispose older Asian women to pupillary block-mediated angle closure [1,2]. In Asian eyes, LPI is also known to be less effective at preventing long-term IOP rise compared with Caucasian eyes [2], further supporting the preference for primary lens extraction in this population.

Our IOP outcomes, a 73.9% reduction from 53.2 to 13.9 mmHg at three months, align closely with published evidence. Husain et al. [6] demonstrated in a randomized controlled trial that PEA+IOL resulted in significantly fewer IOP failures at two years compared with LPI in APAC eyes with coexisting cataract. Importantly, Chan et al. [7] followed this RCT cohort for 10 years and confirmed the durability of this advantage: the phacoemulsification group maintained superior IOP control and required fewer glaucoma medications at 10-year follow-up, with no significant difference in visual field outcomes between groups. This long-term evidence base strongly supports PEA+IOL as the definitive surgical strategy. Recent long-term outcome data from Guo et al. [15] further reinforced this conclusion in a contemporary Chinese cohort undergoing lens extraction for APAC. The narrowing of IOP standard deviation from 15.4 mmHg at baseline to 3.4 mmHg in three months in our series corroborates the normalizing effect of lens extraction on anterior segment dynamics [10].

The superiority of lens-based surgery over LPI for angle-closure disease is further supported by systematic review evidence. Ong et al. [9] conducted a Cochrane systematic review of lens extraction for chronic angle-closure glaucoma and concluded that phacoemulsification reduces IOP and the need for medications more effectively than LPI, with acceptable safety profiles. Brown et al. [10] provided a comprehensive review of lens-based glaucoma surgery, emphasizing that removal of the crystalline lens lowers IOP by deepening the anterior chamber angle and reducing trabecular crowding. For cases complicated by severe refractory APAC with cataract, Hou et al. [16] demonstrated in a randomized clinical trial that although phacotrabeculectomy achieves lower absolute IOP than phacoemulsification alone, the complication rate is higher; phacoemulsification without trabeculectomy remains the preferred approach for most APAC cases.

The mean logMAR VA improvement of 0.95 units is clinically meaningful, representing approximately 9-10 Snellen lines. This is consistent with the long-term outcomes reported by Jeong et al. [17], in which 76% of APAC patients achieved final decimal VA ≥ 0.5, with blindness in only 6.2%. The shift of the modal VA category from CF equivalent at baseline to logMAR ≤ 0 at final follow-up underscores the transformative visual benefit of timely surgical intervention [5,8,16].

The 43.2% rate of postoperative paralytic mydriasis in our cohort is a clinically significant finding that is infrequently quantified in existing literature. Iris sphincter ischemia occurs when IOP remains severely elevated for a prolonged period [1]. With a mean presenting IOP of 53.2 mmHg, many eyes in our series likely exceeded the threshold for irreversible sphincter damage. The resulting mydriasis-causing photophobia, glare, and cosmetic disfigurement cannot be reversed surgically in most cases. Clinicians should counsel patients regarding this risk, particularly when presentation is delayed or IOP is severely elevated [18].

The need for additional glaucoma treatment in 26.6% of eyes is consistent with published rates following primary surgical management of APAC [2,3]. This rate is lower than the 43% reported in a prospective observational series requiring additional medication within one year [3], and notably lower than the up to 56% requiring further intervention after LPI in Caucasian populations [2]. Huang et al. [19] recently identified clinically relevant predictors of glaucoma progression after APAC, including higher presenting IOP, longer duration of attack, and the extent of peripheral anterior synechiae-factors that may help stratify patients who require closer long-term surveillance after surgery. The 5.7% requiring additional surgery (vitrectomy, IOL fixation, or filtering surgery) likely represents cases with extensive peripheral anterior synechiae or concomitant pathology limiting the IOP-lowering effect of lens removal [15,20].

The seasonal distribution of APAC onset has been a topic of long-standing interest because environmental factors such as ambient temperature, humidity, and barometric pressure are hypothesized to influence pupillary block through their effects on iris and lens dynamics. In our 17-year cohort, the temporal distribution of APAC onset did not significantly deviate from a uniform distribution at the α = 0.05 level, either in the monthly (p = 0.095) or seasonal analysis (p = 0.895). Although September showed the highest case count (29 eyes) and June the lowest (10 eyes), this variability did not reach statistical significance. Previously published data from East Asian populations have produced mixed results, with some series suggesting a winter or summer predilection while others have reported uniform distributions similar to our findings. The relatively temperate climate of the Hyogo region in western Japan, with smaller seasonal extremes than those reported in northern Asia, may partly account for the absence of a clear seasonal pattern in our cohort. Our results suggest that, at least in this geographic setting, clinicians should remain vigilant for APAC year-round rather than during specific seasons.

Several limitations of this study should be acknowledged. First, this was a long-term retrospective, single-center study involving multiple ophthalmologists. Consequently, postoperative medications, reoperation, and the follow-up period were determined at the discretion of each treating physician. Second, follow-up was incomplete at the three-month time point (n = 156 of 229 eyes; 68.1%), which may introduce attrition bias toward eyes with favorable outcomes. Third, 19 patients had bilateral involvement, introducing potential intra-patient clustering; although our sensitivity analysis restricting to one eye per patient yielded directionally identical results, a fully clustered approach (e.g., generalized estimating equations or mixed-effects models) was beyond the scope of this descriptive cohort study. Fourth, the absence of a comparison arm (e.g., LPI-first or combined phacotrabeculectomy) precludes direct comparative conclusions [5,6,8], and standardized visual field testing, evaluation of peripheral anterior synechia after cataract surgery, and structural optic-nerve assessment were not uniformly available across the 17-year study period, precluding evaluation of long-term glaucomatous progression. Finally, the assessment of paralytic mydriasis was based on clinical examination and photographic documentation rather than standardized pupillometry, and inter-examiner variability cannot be excluded. Future prospective multicenter studies incorporating standardized gonioscopy, anterior segment optical coherence tomography, nerve fiber layer analysis, automated pupillometry, and patient-reported quality-of-life measures would better characterize the determinants of long-term outcomes [7].

## Conclusions

In conclusion, primary PEA+IOL implantation is a highly effective surgical strategy for APAC in a Japanese elderly population. However, the high rate of postoperative paralytic mydriasis and the need for additional glaucoma treatment underscore the complexity of APAC management and reinforce the importance of long-term surveillance. This study provides significant evidence demonstrating the clinical utility and complication rates of sequential PEA and IOL implantation for APAC.
